# Diabetes, obesity, and insulin resistance in COVID-19: molecular interrelationship and therapeutic implications

**DOI:** 10.1186/s13098-021-00639-2

**Published:** 2021-03-01

**Authors:** Andrey Santos, Daniéla Oliveira Magro, Rosana Evangelista-Poderoso, Mario José Abdalla Saad

**Affiliations:** 1 Department of Internal Medicine-FCM, State University of Campinas-UNICAMP, Campinas, SP Brazil; 2 Department of Surgery, Faculty of Medical Sciences, State University of Campinas-UNICAMP, Campinas, SP Brazil; 3 Faculty of Medical Sciences, State University of Campinas-UNICAMP, Campinas, SP Brazil

**Keywords:** COVID-19, Diabetes, Obesity, Insulin resistance, ISR, iDPP4, Metformin

## Abstract

**Background:**

Our understanding of the pathophysiology of the COVID-19 manifestations and evolution has improved over the past 10 months, but the reasons why evolution is more severe in obese and diabetic patients are not yet completely understood.

**Main text:**

In the present review we discuss the different mechanisms that may contribute to explain the pathophysiology of COVID-19 including viral entrance, direct viral toxicity, endothelial dysfunction, thromboinflammation, dysregulation of the immune response, and the renin–angiotensin–aldosterone system.

**Conclusions:**

We show that the viral infection activates an integrated stress response, including activations of serine kinases such as PKR and PERK, which induce IRS-1 serine phosphorylation and insulin resistance. In parallel, we correlate and show the synergy of the insulin resistance of COVID-19 with this hormonal resistance of obesity and diabetes, which increase the severity of the disease. Finally, we discuss the potential beneficial effects of drugs used to treat insulin resistance and diabetes in patients with COVID-19.

## Introduction

The COVID-19 outbreak, caused by the novel coronavirus (named severe acute respiratory syndrome (SARS) coronavirus 2 (SARS-CoV-2), was first detected at the end of 2019 in Wuhan, China, reached pandemic status in February 2020, and now is present in every country around the world. Our understanding of the pathophysiology of the disease manifestations has improved but the reason why the evolution is more severe in some patients is still unknown. Data coming from different sources has shown that body mass index (BMI) and metabolic syndrome are strong independent risk factors for severe COVID-19 [1, 2]. In the present review we discuss the different mechanisms that may contribute to explain the pathophysiology of COVID-19 including viral entrance, direct viral toxicity, endothelial dysfunction, thromboinflammation, dysregulation of the immune response, and the renin–angiotensin–aldosterone system (RAAS) [1, 2]. Some of these mechanisms are common to sepsis such as the release of cytokines and microcirculation dysfunction, others are specific to COVID-19 such as Angiotensin-converting enzyme 2 (ACE2) mediated viral entry and tissue damage, and dysregulation of the RAAS [3]. In parallel, we correlate these mechanisms with obesity, diabetes, and insulin resistance, which increase the severity of the disease, and the potential beneficial effect of drugs used to treat insulin resistance and diabetes in patients with COVID-19.

## Pathophysiology

### Viral entrance: ACE2 and DPP4

**Fig. 1:**
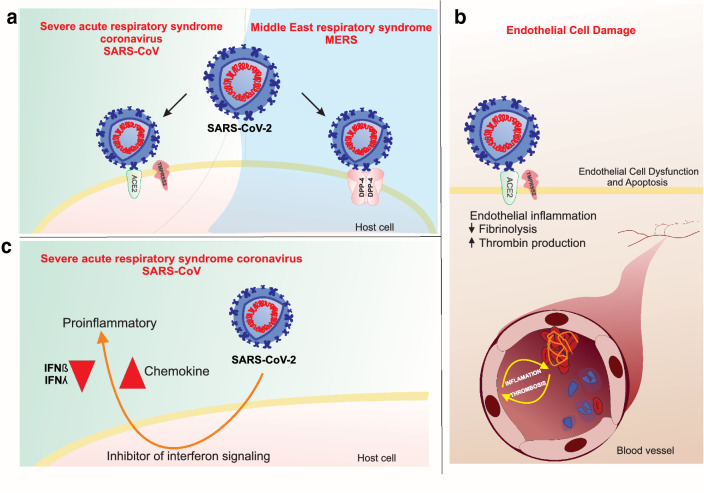
Pathophysiology. **a **Viral entrance: ACE2 and DPP4. The coronavirus entrance in cells is facilitated by its spike protein using ACE2. **b **Endothelial cell damage and thromboinflammation. ACE2-mediated entry of SARS-CoV-2 in endothelial cells induce inflammation and the generation of a prothrombotic milieu, and results in increased thrombin production associated with inhibition of fibrinolysis and activation of complement pathways, a cascade which will lead to microthrombi deposition. The cross-talk between platelets and neutrophils and the activation of macrophages has an important role in the proinflammatory effects, characterized by cytokine release, the formation of neutrophil extracellular traps (NETs), and microthrombus deposition. **c **Dysregulation of the immune response. Dysregulation of the immune response of COVID-19, in which there is an increase in cytokine release associated with an attenuation of interferon response

The coronavirus entrance into cells is facilitated by its spike protein using ACE2 as an entry receptor, with higher affinity binding. In this process the binding of the spike protein by the cellular serine protease TMPRSS2 [4] is also important, indicating that both ACE2 and TMPRSS2 are key proteins in the process for the virus entrance (Fig. 1a). This is the most accepted mechanism of entrance, but some other considerations have also appeared. Although DPP4 was initially described as receptor for SARS-CoV, which induced Middle East respiratory syndrome (MERS), recently an affinity between DPP4 and the spike (S) receptor-binding domain of SARS-CoV-2 was shown by a bioinformatics approach, indicating that DPP4 is also a potential binding target for SARS-CoV-2 (Fig. 1a).

In addition to upper and lower airways, SARS-CoV-2 also has tropism to renal [5, 6], myocardial [7], neurologic and gastrointestinal [8] tissues, associated with expression of ACE2 and TMPRSS2 in these tissues [9–11], suggesting that direct viral tissue damage may occur in these tissues.

### Endothelial cell damage and thromboinflammation

It is now well established that ACE2-mediated entry of SARS-CoV-2 into endothelial cells induces inflammation and the generation of a prothrombotic milieu [12–14] (Fig. 1b). These alterations in the set of COVID-19 result in increased thrombin production associated with inhibition of fibrinolysis and activation of complement pathways, a cascade which will lead to microthrombi deposition [14–17]. The cross-talk between platelets and neutrophils and the activation of macrophages have an important role in the proinflammatory effects, characterized by cytokine release, the formation of neutrophil extracellular traps (NETs), and microthrombus deposition [18–22]. Furthermore, acute lung injury can activate a mechanism related to hypoxia-induced hyperviscosity associated with an increase in HIF-1 (hypoxia-inducible factor 1) signaling pathway, which can synergically increase the prothrombotic state[1, 23].

### Dysregulation of the immune response

Dysregulation of the immune response is a key characteristic of COVID-19, in which there is an increase in cytokine release associated with an attenuation of interferon response, mainly due to T cell lymphodepletion [24] in parallel with a hyperactivations of innate immunity (Fig. 1c).

The type I and type II interferon receptors are expressed in immune cells and other cell types, and type III interferon receptor is expressed in epithelial cells in the upper and lower respiratory airway. Type I and type II interferons produce a pro-inflammatory response and type III interferons reduce viral replication, induce epithelial barrier stability, and are less inflammatory. In this regard, it is easy to understand why an appropriate interferon response is essential to combat a virus and eliminate a viral infection.

In older persons, in obesity and diabetes there is an impaired early antiviral interferon response, contributing to susceptibility to severe COVID-19 in these individuals. In addition, SARS-CoV-2 infection also potentiates the existing chronic inflammatory state resulting in severe disease.

**Fig. 2:**
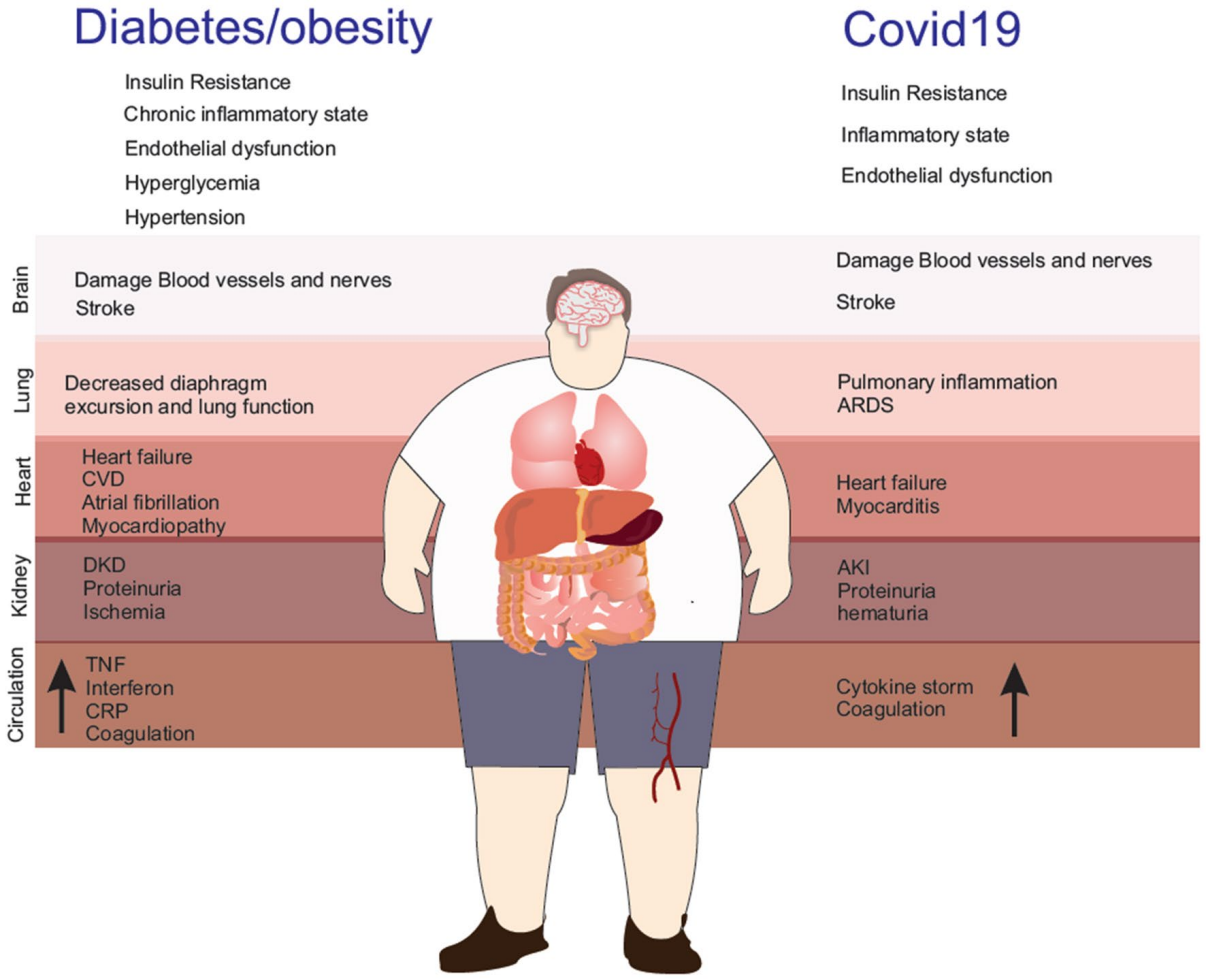
Clinical manifestations and complications of this insulin resistance syndrome and the parallel with obesity/diabetes. T2D and obesity, without infections, have an increase in the basal inflammatory response and a potential decrease in an interferon response. Thus, the synergy between COVID-19 and T2D/obesity may amplify the inflammatory response and downregulate even more the interferon response, contributing to more severe disease in these patients

In other words, in parallel with attenuation of interferon signaling there is a clear activation of neutrophils and monocyte-macrophages which are the main mediators of hyperinflammation [25, 26]. Neutrophils have a key role in the pathogenesis of COVID19, because SARS-CoV-2 induces infiltration of these cells in the lungs. Necrotic products of cell death as well as neutrophil extracellular traps (NETs) produced during infection activate pattern recognition receptors (PRR), aggravating the cytokine storm [27, 28]. In addition, NETs can contribute to endothelial dysfunctions and venous thrombosis. In diabetes these effects are potentiated, because hyperglycemia triggers neutrophils to release NETs which in turn contribute to the cytokine storm and sepsis in COVID-19 [29]. This inflammatory state is demonstrated by an increase in serum inflammatory markers such as erythrocyte sedimentation rate, C-reactive protein, ferritin, fibrinogen, D-dimer, and lactate dehydrogenase. Higher levels of these laboratory markers are predictive of subsequent critical illness and mortality in patients with COVID-19 [30–34]. The manifestations related to the immune system and cytokine-release syndrome presented in patients with COVID-19 are illustrated in Fig. 2.

In summary, severe COVID-19 may have a dual pathophysiology: an initially poor antiviral interferon response and an exaggerated inflammatory response, which is not efficient to eliminate the virus. Moreover, diabetes mellitus type 2 (DM2) and obesity, without infections, have an increase in the basal inflammatory response and a potential decrease in an interferon response. Thus, the synergy between COVID-19 and DM2/obesity may amplify the inflammatory response and downregulate even more the interferon response, contributing to more severe disease in these patients (Fig. 2).

There are also modulations of cellular immunity in COVID-19, which can have important clinical implications for long-term immunity. During the acute phase, T cells develop a cytotoxic phenotype that correlates with some clinical and laboratory markers of disease severity described above. In contrast, the convalescent phase of COVID-19 specific T cells behaves as polyfunctional, associated with a stem-like memory phenotype. These data indicate that SARS-CoV-2 elicits a clear and important functional memory T cell response, suggesting permanent immunity after natural exposure (cell recent) [32–36].

### Dysregulation of the RAAS

Modulations of the RAAS are another piece of the puzzle of the pathophysiological mechanism of COVID-19. The RAAS can be summarized as a cascade of regulatory peptides, peptidases, and receptors that participate in many homeostatic processes of the body, including blood pressure regulation, fluid and electrolyte balance, vascular permeability, and tissue growth [37].

**Fig. 3:**
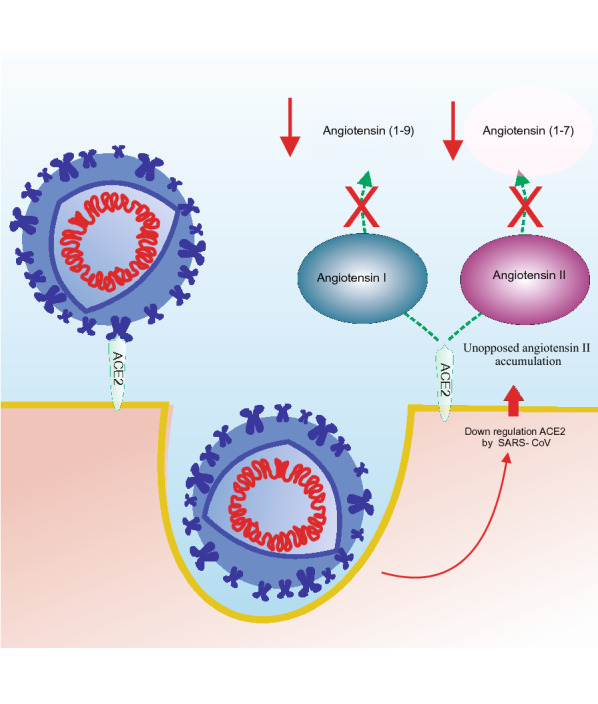
Dysregulation of the RAAS. ACE2 cleaves AI and AII into inactive angiotensin 1–9 and angiotensin 1–7, respectively, and the later has vasodilator and antifibrotic effects, but SARS-CoV binds and downregulates the expression of ACE2, which is associated with the unopposed AII action

Angiotensin-converting enzyme (ACE) converts angiotensin I (AI) into angiotensin II (AII), which induces vasoconstriction and proliferation. Although ACE2 is not canonically mentioned in this system, this enzyme is a membrane-bound aminopeptidase that counteracts the RAAS pathway. ACE2 cleaves AI and AII into inactive angiotensin 1–9 and angiotensin 1–7, respectively, and the later has vasodilator and antifibrotic effects (Fig. 3).

In experimental diabetes there is an increase in ACE expression in different tissues, which can explain why patients with diabetes may have more severe disease. It is important to mention that the ratio of ACE/ACE2 is increased in the lungs of patients with ARDS, favoring AII generation. SARS-CoV binds and downregulates the expression of ACE2, which is associated with the unopposed action of AII which can have a critical role in lung injury. These data suggest that reducing the ACE/ACE2 ratio may potentially have beneficial effects in lung injury in COVID-19. In this regard many drugs can modulate this ratio with therapeutic implications for patients with obesity and diabetes: insulin reduces ACE2 expression, but GLP-1 agonists, pioglitazone, ACE inhibitors, and statins upregulate ACE2, reducing the ACE/ACE2 ratio [38–44].

In summary, ACE2 is considered a counter-regulator against ACE because it converts angiotensin II (Ang-II) to Ang-(1–7), preventing the increase in AII. SARS-CoV binds and downregulates the expression of ACE2 and Ang-(1–7), which might increase AII. It is well known that AII has important inflammatory activity, increasing the migration of monocytes and also the infiltration of macrophages [45]. In addition, this leads to loss of protection of endothelial function, because a decrease of counter-regulation of angiotensin II signaling is followed by vascular inflammation and thrombosis of peripheral blood vessels.

### Molecular mechanism of insulin resistance in COVID-19: integrated stress response (ISR)

**Fig. 4:**
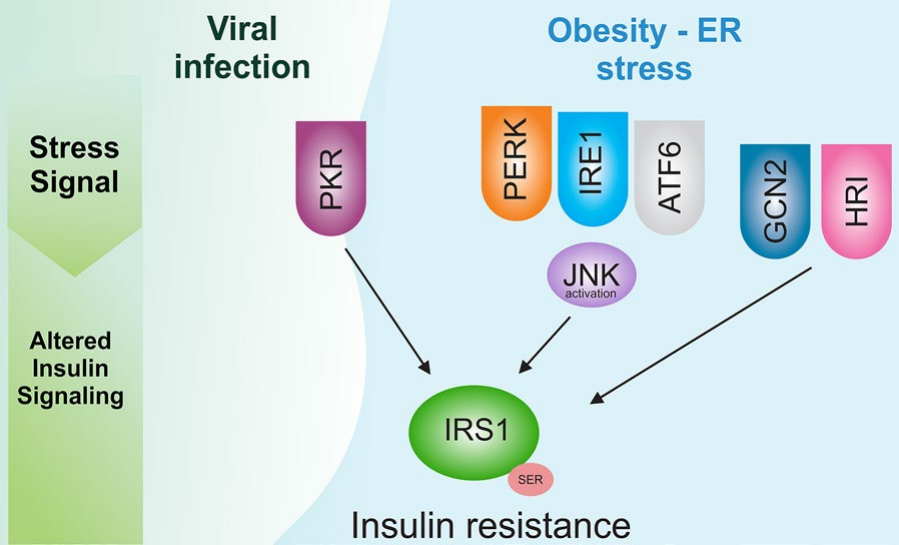
Integrated stress response (ISR). The ISR signaling pathway initiates when distinct stressors activate at least one member of a family of four serine/threonine kinases—PERK, PKR, HRI, GCN2, and these activations will converge to induce phosphorylation of eIF2a on serine. At least two of the four serine/threonine kinases activated by distinct stressors—PKR and PERK—can downregulate the insulin signaling pathway through serine phosphorylation of insulin receptor substrates, attenuating insulin action. Specifically for COVID-19, fragments of viral RNA can activate PKR, which will induce IRS-1 serine phosphorylation and consequently insulin resistance

Recently, stress response at the cellular level was re-organized in a convergent signaling pathway called integrated stress response (ISR), which can be activated by multiple physiological and pathological situations or stressors, including hypoxia, viral infection, and cell-intrinsic stresses such as endoplasmic reticulum (ER) stress. The ISR signaling pathway initiates when distinct stressors activate at least one member of a family of four serine/threonine kinases—PKR-like ER kinase (PERK double-stranded RNA-dependent protein kinase (PKR), heme-regulated eIF2a kinase (HRI), and general control non-derepressible 2 (GCN2)—(Fig. 4) [46–48] and these activations will converge to induce phosphorylation of eIF2a on serine. A decrease in protein synthesis is prompted by elF2 phosphorylation and at the same time induces the translation of selected genes, promoting cell survival and recovery [49], but the final response depends on whether the cellular stress is severe or not. If the cellular stress is severe the capacity of the adaptive response to resolve it will be overwhelmed, and other components will be activated to induce cell death. Dephosphorylation of eIF2a blocks the ISR, normaling the synthesis of the PKR gs2 protein [50–52].

As described, during this ISR, at least two of the four serine/threonine kinases activated by distinct stressors—PKR and PERK—can downregulate the insulin signaling pathway through serine phosphorylation of insulin receptor substrates, attenuating insulin action [53–55]. Specifically for COVID-19, fragments of viral RNA can activate PKR, which will induce IRS-1 serine phosphorylation and consequently insulin resistance. We therefore suggest that insulin resistance always accompanies ISR. Moreover, the cytokine storm and also an increase in hormonal signaling such as AII and cortisol can activate some of the four kinases and contribute to insulin resistance [56]. The clinical manifestations and complications of this insulin resistance syndrome and the parallel with obesity/diabetes are described in Fig. 2.

### Insulin resistance induced inflammation

**Fig. 5:**
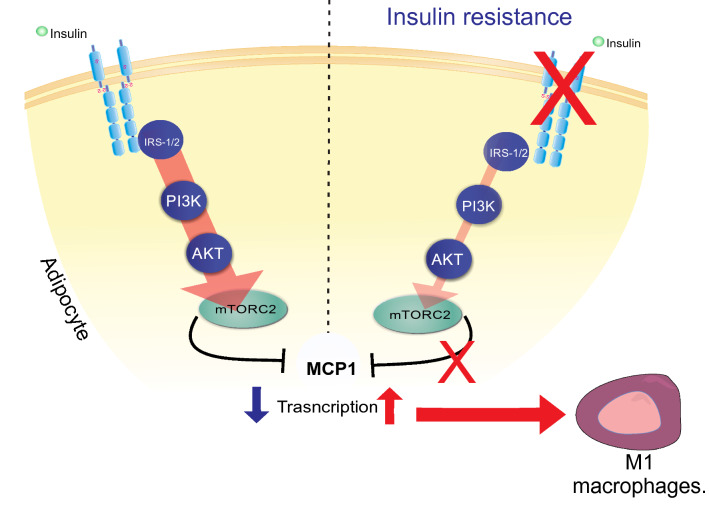
Insulin resistance induced inflammation. Insulin resistance in adipose tissue induces macrophage infiltration, developing an inflammatory state. In the insulin signaling pathway (IRS-1/2:PI3K:AKT:mTORC2) the MCP1 gene is suppressed by mTORC2 in adipose. In situations of insulin resistance, the downregulation of insulin signaling with reduced activity of mTORC2 will derepress MCP1 and will attract monocytes to adipose tissue, which will be converted into M1 macrophage

Although it is well accepted that inflammation in obesity can induce insulin resistance, recent evidence suggests that the opposite is also true, i.e., insulin resistance by itself can also induce inflammation [57]. Insulin resistance in adipose tissue induces macrophage infiltration, developing an inflammatory state. The molecular mechanisms for this relationship are just beginning to be unraveled, but it is important to mention that in the insulin signaling pathway, towards AKT activation there is a serine–threonine protein kinase complex called mTORC2, which is activated by AKT and is an important mediator of insulin action in glucose metabolism and gene expression. One important gene suppressed by mTORC2 in adipose is monocyte chemoattractant protein 1 (MCP1). In this regard, in situations of insulin resistance, the downregulation of insulin signaling with reduced activity of mTORC2 derepresses MCP1 and will attract monocytes to adipose tissue, which will be converted into M1 macrophage [57, 58] (Fig. 5). This data shows that the “chicken-and-egg” relationship of insulin resistance and inflammation is not yet solved. We can thus suggest that inflammation induced by insulin resistance may also be an aggravating mechanism to the cytokine storm observed in Covid-19 patients with obesity.

### Insulin resistance, hyperinsulinemia and lung mechanics

**Fig. 6:**
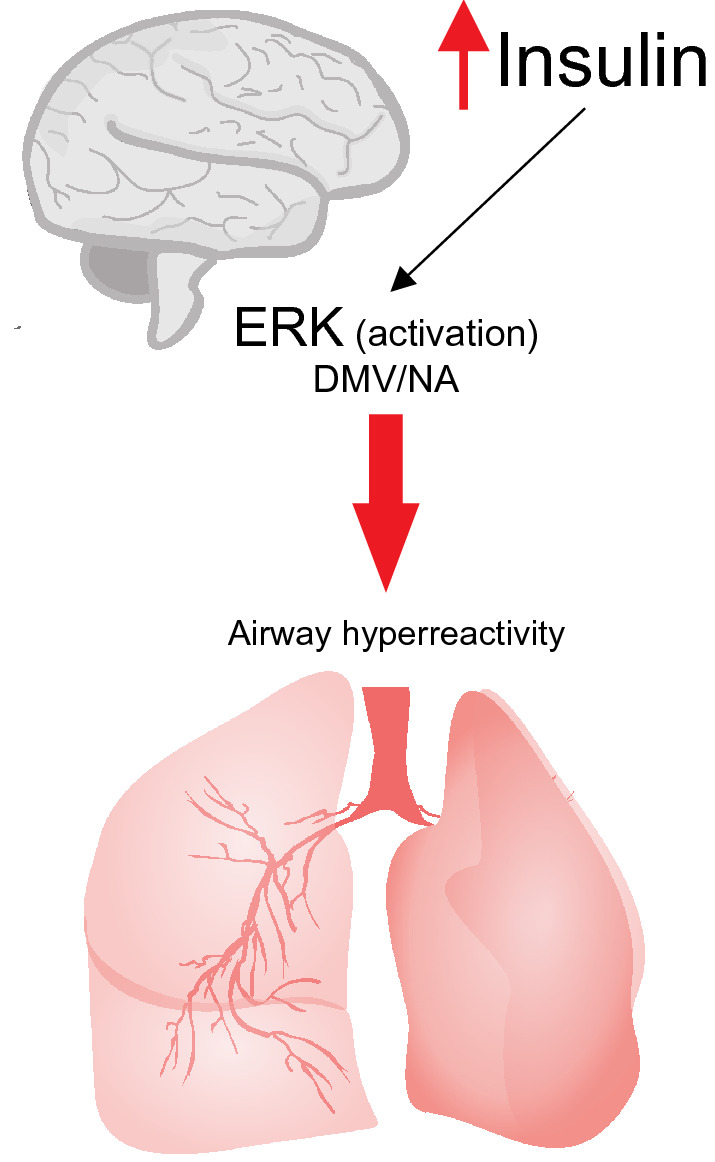
Airway hyperreactivity. The central insulin action is able to control airway reactivity, and in obesity central hyperinsulinemia induces an increased airway hyper-reactivity by stimulating airway-related pre-ganglionic parasympathetic fibers at the dorsal motor nucleus of the vagus (DMV) and nucleus ambiguous (NA), by the ERK signaling pathway

Insulin resistance induces dramatic consequences for health, affecting vessels, heart, brain, and kidneys, however the effects of this hormonal resistance on lung function is only marginally known. Previous data has shown that obesity and fat accumulation in the abdomen pushes up the diaphragm and restricts airflow. Reduced lung volumes subsequently lead to collapse of airways in the lower lobes of the lung. This mechanical mechanism is important, but certainly is not the only one. Our laboratory investigated the effect of insulin resistance on mechanical pulmonary parameters and function in animal models of obesity. The consequences of insulin resistance in obesity and DM2 may be secondary to three main mechanisms: chronic inflammation, lack of insulin effect, or hyperinsulinemia [48]. The results of our investigations of lung function in obesity indicated that hyperinsulinemia is the main driver of lung dysfunction. Our results showed that central insulin action is able to control airway reactivity, and in obesity central hyperinsulinemia induces an increased airway hyper-reactivity by stimulating airway-related pre-ganglionic parasympathetic fibers at the dorsal motor nucleus of the vagus (DMV) and nucleus ambiguus (NA). This insulin effect can theoretically be mediated by PI3K/Akt or via ERK signaling, and our results showed that the later pathway accounts completely for this insulin action. It is important to mention that insulin is able to increase all lung mechanical parameters, including tissue resistance (Gtis) and tissue elastance (Htis), indicating that central insulin action modulates both the more calibrous bronchial branches and the very distal bronchioles and lung tissue. We also demonstrated that hyperinsulinemia, independent of body weight, is able to induce bronchoconstriction per se. In summary our data showed that hyperinsulinemia causes airway hyperreactivity in obese mice, through activation of cholinergic nerves in the brain stem, by the ERK signaling pathway. These data have important clinical implications, because in obesity, altered lung mechanics was usually attributed mainly to abdominal compression of the lung, but our data showed that hyperinsulinemia may also have a critical role (Fig. 6).

### Similarities and synergy between insulin resistance/obesity/diabetes and COVID-19

**Fig. 7:**
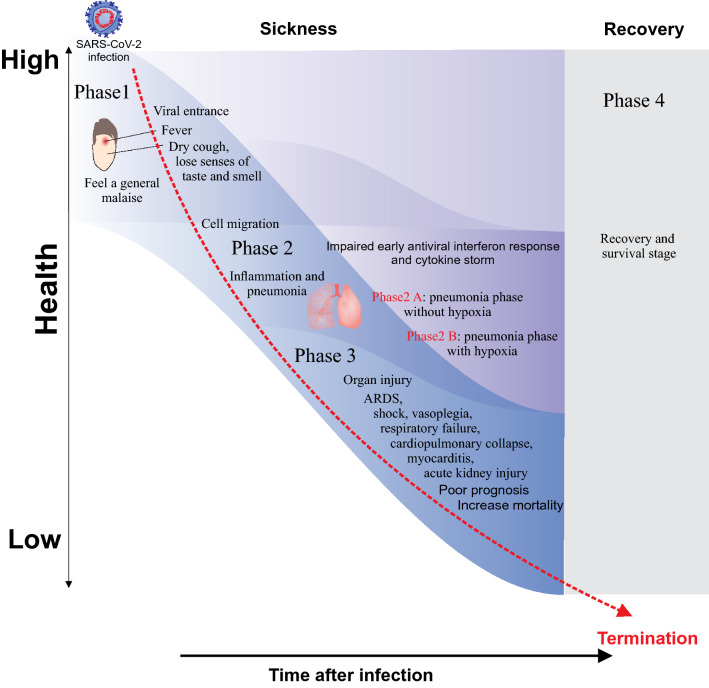
Similarities and synergy between insulin resistance/obesity/diabetes and COVID-19. COVID-19. In symptomatic patients with COVID-19 the course of the disease can be didactically divided into four phases. Phase 1: Viral entrance in cells is facilitated by ACE2. Phase 1 starts when an individual becomes symptomatic. The most frequent manifestations in this phase are fever and dry cough, and many individuals may lose their senses of taste and smell and feel a general malaise, and for most individuals, the disease is limited to this phase. Phase 1/Phase 2: Immune cell migration to the lungs. Phase 2: Is the pulmonary stage of the disease when individuals develop pulmonary inflammation and pneumonia. In this phase there is an impaired early antiviral interferon response and cytokine storm. Based on the presence or not of hypoxia this phase can be subdivided in 2b or 2a. Most individuals need hospitalization, and some with prolonged hypoxia need mechanical ventilation. Phase 3: the patients develop ARDS and extrapulmonary systemic hyper inflammation syndrome, shock, vasoplegia, respiratory failure, cardiopulmonary collapse, myocarditis, and acute kidney injury, with poor prognosis and increased mortality. Phase 4: Is the recovery and survival stage

In symptomatic patients with COVID-19 the course of the disease can be didactically divided into four phases (Fig. 7). Phase 1 starts when an individual becomes symptomatic [59]. The most frequent manifestations in this phase are fever and dry cough, and many individuals may lose their senses of taste and smell and feel a general malaise, and for most individuals, the disease is limited to this phase [59]. Phase 2 is the pulmonary stage of the disease [59] when individuals develop pulmonary inflammation and pneumonia. Based on the presence or not of hypoxia this phase can be subdivided into 2b or 2a [59]. Most individuals need hospitalization, and some with prolonged hypoxia need mechanical ventilation [59]. When there is progression to phase 3 the patients develop ARDS and extrapulmonary systemic hyper inflammation syndrome, shock, vasoplegia, respiratory failure, cardiopulmonary collapse, myocarditis, and acute kidney injury [59], with poor prognosis and increased mortality. Finally, phase 4 is the recovery and survival stage [60] (Fig. 7).

Data coming from different sources show that loss of metabolic health is the most important risk factor for severe COVID-19, and in this regard DM2, obesity, and hypertension are critical comorbidities in people with COVID-19 [61–63]. There are no simple explanations for these findings which most probably are multifactorial. There are clear similarities and/or synergy between DM2 and COVID-19 related to mechanism and complications, as described in Fig. 2. At this point it is important to emphasize that metabolic syndrome and DM2 are accompanied by a low chronic inflammatory state, and since COVID-19 also presents a hyperinflammatory response, a synergy between these inflammatory situations may increase the damage induced by inflammatory mediators. In the same line, diabetes and hypertension are risk factors for kidney disease. COVID-19 can also result in kidney injury and patients with diabetes and hypertension are more susceptible to kidney damage during COVID-19 infection. Many other similarities and synergies are presented in Fig. 2.

### Hyperglycemia and endocrine pancreas

Previous data in diabetic patients at hospital admission showed that hyperglycemia was an important predictor of worse outcomes such as ICU admission, mechanical ventilation and death [64, 65]. Similar results were observed in in-hospital glycemic control, i.e., higher blood glucose levels and bad metabolic control were also associated with worse outcome [66–68]. It is important to mention that SARS-Cov2 can not only impair diabetes control, but can account for some new cases of diabetes. In this regard, this virus tropism for the β-cell can certainly impair insulin secretion, but can also lead to destruction of β-cells, inducing new-onset diabetes [69, 70].

The localization of ACE2 expression in the endocrine part of the pancreas suggests that SARS coronavirus enters islets using ACE2 as its receptor and damages islets causing acute diabetes [71, 72]. However, this is not uniformly accepted because the preferred target for islet viral infection appears to be microvascular structures and ducts, rather than endocrine cells. Regarding the expression of ACE2 in the islets, the studies are contradictory regarding an increase in its expression during the infection of SARS-CoV-2. It seems that this contradiction can occur due to the difficulty of isolating the islets that can affect the expression of ACE2 [71, 73, 74]

## Therapeutical implications for metabolic syndrome or diabetes in patients with COVID-19 (Fig. 8)

**Fig. 8:**
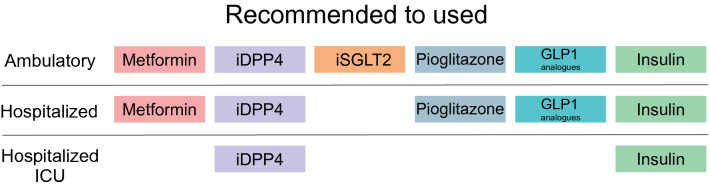
Diabetes treatment in patients with COVID-19. There is no reason to stop metformin therapy during COVID-19 infection unless there are severe gastrointestinal symptoms, or risk of lactic acidosis. Metformin can be a beneficial adjuvant therapy for patients in acute, chronic, and even recovery phases of COVID-19. The continued use of sulfonylurea in stable patients with COVID-19 who are eating regular meals may be justified. However, we need to be alert to any potential risks of hypoglycemia, especially in patients with COVID-19 in intensive care units. A theoretical anti-viral effect of SGLT2-inhibitors was suggested, however caution should be taken when using these drugs because they require hydration and appropriateness of insulin doses to prevent euglycemic ketoacidosis. GLP-1 receptor agonists should be carefully evaluated in severely ill patients with COVID-19 considering their anorexic effects. However, their potential beneficial effects should also be balanced, because these drugs have anti-inflammatory and lung protection actions and can be valuable weapons to combat COVID-19. DPP-4 inhibitors are a group of drugs associated with many advantages, even in severe cases of COVID-19, because they are well tolerated, can be used independent of renal function, and have a low risk of hypoglycemia. In this regard, we should consider recommending a more widespread use of DPP4 inhibitors in diabetic inpatients with severe COVID-19. In most studies, DM2 patients with COVID-19 on insulin have shown a worse prognostic, usually attributed to the severity of diabetes in these patients. However, by lowering the doses of insulin, by association with oral anti-hyperglycemic agents, it is possible to attenuate this potential worse effect of high doses of insulin

The treatment of obese and/or diabetic patients with COVID-19 deserves special considerations, based on recent clinical experience associated with better understanding of pathophysiological mechanisms. For mild COVID-19 the oral glucose-lowering therapies for patients with diabetes could be maintained. In contrast, for inpatients with diabetes and severe COVID-19 the initial indications were to withdrawing ongoing treatments and initiating insulin therapy. It is important to mention that this decision is not a consensus anymore and should be based on the same considerations undertaken for inpatients with diabetes, including severity of associated diseases, nutritional status, glycemic control, and risk of hypoglycemia, hepatic, cardiac, and renal function.

In most studies, DM2 patients with COVID-19 on insulin have shown a worse prognostic, usually attributed to the severity of diabetes in these patients. However, as previously described in experimental models intracerebroventricular-injected insulin increases airway reactivity, lung resistance, and elastance [48]. Thus, it is tempting to speculate that hyperinsulinemia in obesity and diabetes can impair airway reactivity, and that administration of high doses of insulin can worsen this effect, but by lowering the doses of insulin, by association with oral anti-hyperglycemic agents, it is possible to attenuate this potential worse effect of high doses of insulin.

A recent study has shown better clinical outcomes in patients on metformin compared with those on insulin, but again we should consider that the use of insulin may just indicate that the diabetes was more severe and should be interpreted as a potential confounder. Preliminary retrospective data have shown a reduction in death rates in DM2 with COVID-19 hospitalized patients using metformin compared with non-users [75]. Although definitive conclusions can be obtained only by RCTs, based on previous data we can suggest that there is no reason to stop metformin therapy during COVID-19 infection unless there are severe gastrointestinal symptoms, or risk of lactic acidosis. The mechanism by which metformin may protect patients with DM2 and COVID-19 has been attributed at least in part to the effect of the drug that might prevent virus entry into target cells via AMPK activation and the PI3K/Akt signaling pathway [76]. Additionally, metformin also has anti-inflammatory effects that might prevent the cytokine storm [77].

Furthermore, in pre-clinical studies metformin has been shown to improve lung fibrosis, suggesting that this drug should be investigated in the treatment of COVID-19-related pulmonary fibrosis [78, 79]. Taken together, all these data support the opinion that metformin can be a beneficial adjuvant therapy for patients in acute, chronic, and even recovery phases of COVID-19.

Sulphonylureas are associated with an increased risk of hypoglycemia, which can be severe and prolonged [80]. The continued use of sulfonylurea in stable patients with COVID-19 who are eating regular meals may be justified. However, there is an association of sulfonylureas with an increased risk of hypoglycemia, especially in ICU patients with limitations on food intake. In addition, as COVID-19 can cause cardiac injury, caution is recommended in the use of sulfonylureas that have the nonselective binding of sulfonylureas to pancreatic and cardiac sulfonylurea receptors [81, 82]. Newer drugs such as gliclazide and glimepiride that selectively bind to pancreatic receptors may be safer [83]. Randomized clinical trials are needed to outline a guideline. Clearly, we need to be alert to any potential risks of hypoglycemia, especially in patients with COVID-19 in intensive care units.

A theoretical anti-viral effect of SGLT2-inhibitors (iSGLT2) was suggested, considering that these drugs can increase lactate concentrations and decrease intracellular pH, reducing the viral load [84]. Caution should be taken when using these drugs because they require hydration and appropriateness of insulin doses to prevent euglycemic ketoacidosis.

GLP-1 receptor agonists should be carefully evaluated in severely ill patients with COVID-19 considering their anorexic effects. However, their potential beneficial effects should also be balanced, because these drugs have anti-inflammatory and lung protection actions and can be valuable weapons to combat COVID-19. The anti-inflammatory effects of GLP-1 agonists have been demonstrated in DM2 patients, through reduction in the expression of TNFα, IL-1β, JNK-1, and decreased IL-6 concentrations. In addition, in endothelial cells GLP-1 agonists are able to reduce the generation of reactive oxygen species, improving endothelial dysfunction of COVI-19.

DPP-4 inhibitors are a group of drugs associated with many advantages, even in severe cases of COVID-19, because they are well tolerated, can be used independent of renal function, and have a low risk of hypoglycemia. In addition, experimental studies, demonstrated that these drugs can reduce inflammatory response. We now believe that iDPP4 are very safe and can potentially be beneficial to most inpatients with diabetes and COVID-19 for the following reasons: (A) Recently, pooled data from three prospective studies in inpatients with DM2 showed that treatment with iDPP4 alone or in combination with basal insulin is effective, and results in a lower incidence of hypoglycemia and in the use of lower doses of insulin compared to a basal bolus insulin regimen [85]. (B) In addition, recent data has demonstrated an affinity between DPP4 and the spike (S) receptor-binding domain of SARS-CoV-2, by a bioinformatics approach, indicating that DPP4 is a potential binding target for SARS-CoV-2 [86]. This study points to DPP4 as a binding target for SARS-CoV-2 and reinforces DPP4 as a very promising target to attenuate COVID-19 severity.

Finally, there are now six studies [2, 87–91] (Table 1) that have investigated the effects of DPP4 inhibitors on the outcomes of COVID-19 in diabetic patients. Three retrospective studies had a small number of patients on iDPP4, but despite this, Rhee et al. [90] demonstrated that the fatality rates and ventilation scores of patients treated with DDP4i were lower than those of diet alone treated DM2 patients. The Coronado study showed no independent association between severity of COVID-19 and use of DPP4 inhibitors, but we should emphasize that they only considered iDPP4 use prior to admission. Recently, in a preprint manuscript Rhee et al. showed a significant beneficial effect of iDPP4 on primary outcomes. More importantly, very recently an Italian group showed in a case-control study that in patients with type 2 diabetes, sitagliptin treatment at the time of the hospitalization reduced mortality and improved clinical outcome [92]. Although only a randomized trial can definitively answer this question (use of DPP4 inhibitors in patients with COVID-19), based on molecular, pathophysiological, and retrospective studies, the use of iDPP4 is safe, and is certainly beneficial in metabolic control and can probably reduce the severity of COVID-19. In this regard, we should consider recommending a more widespread use of DPP4 inhibitors in diabetic inpatients with severe COVID-19.

**Table 1:** Clinical studies that reported DPP-4 inhibitors in patients with T2DM and COVID-19

First author; year; Country	Simple size	T2M (n.%)	DPP-4 i (n.%)	Age (years)^a^	Male (%)	Outcomes	In-hospital death (n.%)
*Retrospective studies*							
Chen et al. 2020. China [87]	904	136 (15.0)	20 (14.7)	66.0 (56.0–73.0)	NR	Hospital stay (days) 22 (19.3–29.0)	5 (25.0)
Xu et al. 2020. China [88]	364	114 (31.3)	7 (6.1)	66.0 (57–73)	54.4	Lower ventilation score	7 (100.0)
Zhu et al. 2020. China [89]	7337	952 (12.9)	55 (6.8)	NR	NR	20% well controlled	–
Rhee et al. 2020. South Korea [90]	5080	832 (16.3)	263 (31.6)	63.69 (12.2)	56.65	Intensive care 3.42%	4.39
*Multicenter observational study*							
Cariou et al. 2020. France [2]	1317	1166 (88.5)	285 (21.6)	69.8 (13.0)	64.9	Tracheal intubation and/or death within 7 days of admission OR 1.01 (0.75, 1.34)	OR 0.85 (0.55, 1.32)
*Case–control study*							
Fadini et al. 2020 Italy [91]	403	85 (21.1)	9 (10.6)	72.2 (12.8)	77.8	ICU admittance 33.3% Semi-intensive care 44.4%	11.1

*NR* No reported, *ICU* intensive care unit

^a^Age was reported as range; median ± SD

It is well established that peroxisome proliferator-activated receptor-gamma (PPAR-γ) is abundantly expressed in immune cell macrophages and is a blocker of macrophage activation and T cell responses [93]. Pioglitazone, a PPAR-γ ligand, through action in monocytes and macrophage, is able to reduce the secretion of TNF-α, IL-1β, and IL-6 [94]. This drug reduces lung injury and decreases mortality from sepsis by reducing inflammatory cytokine production. In addition, pioglitazone upregulates ACE2 expression, supporting the control of ongoing inflammation, as previously described [95].

It is important to mention that the anti-inflammatory and glucose lowering activities of pioglitazone are two distinct effects induced by different pathways. In this regard, a low dose of pioglitazone is able to reduce TNFα and IL-6 levels without side effects [96]. We can thus speculate that 15 mg of pioglitazone seems to be a treatment option to produce anti-inflammatory effects for COVID-19 patients.

In conclusion, this review presents different mechanisms that may contribute to explain the pathophysiology of COVID-19 including viral entrance, direct viral toxicity, endothelial dysfunction, thromboinflammation, dysregulation of the immune response, and the renin-angiotensin-aldosterone system. Moreover, we show that the viral infection activates an ISR, including activations of serine kinases such as PKR and PERK, which will induce IRS-1 serine phosphorylation and insulin resistance. In parallel, we correlate and show synergy of the insulin resistance of COVID-19 with this hormonal resistance of obesity and diabetes, which increase the severity of the disease (Table 2). Finally, we discuss the potential beneficial effect of drugs used to treat insulin resistance and diabetes in patients with COVID-19, emphasizing the beneficial role of metformin, iDPP4, and pioglitazone (Fig. 8)

**Table 2:** COVID-19 mortality according diabetes or without diabetes

	Study type	Population (n.)	With diabetes n. (%)	Covid-19 mortality		
				With diabetes n. (%)	Without diabetes n. (%)	P-value or RR*
Zhang et al. [97]	Retrospective	258	63 (24)	7 (11.1)	8 (4.1)	0.039
Zhou et al. [30]	Retrospective	191	36 (19)	17 (31)	19 (14)	0.0051
Zhu et al. [89]	Retrospective	7337	952 (13)	74 (7.8)	174 (2.7)	< 0.001
Yan et al. [98]	Retrospective	193	48 (25)	39 (81.3)	69 (47.6)	< 0.001
Barron et al. [99]	Cohort-study	61,414,470	2,864,670 (4.7)	7434 (31.4)	15,831 (0.02)	2.03 (1.97–2.09)*

RR* relative risk

## Data Availability

Not applicable.
